# The Intersection of Childcare and Health Among Women at a U.S. Safety-Net Health System During the COVID-19 Pandemic: A Qualitative Study

**DOI:** 10.1089/heq.2023.0068

**Published:** 2024-01-12

**Authors:** Seema Jain, Robin T. Higashi, Carolina Salmeron, Kavita Bhavan

**Affiliations:** ^1^ HonorHealth Internal Medicine Residency, Phoenix, Arizona, USA.; ^2^ The University of Texas Southwestern Medical Center, Dallas, Texas, USA.; ^3^ UTHealth School of Public Health, Houston, Texas, USA.; ^4^ Parkland Health and Hospital System, Dallas, Texas, USA.

**Keywords:** caregiver health, qualitative research, social determinants of health, social domains of health

## Abstract

**Introduction:**

Lack of childcare has been linked to missed health care appointments for adult women, especially for lower-income women. The COVID-19 pandemic created additional stressors for many low-income families that already struggled to meet childcare and health care needs. By exploring the experiences of women who were referred for childcare services at a U.S. safety-net health system, we aimed to understand the challenges women faced in managing their health and childcare needs during the COVID-19 pandemic.

**Methods:**

We conducted semistructured interviews with participants in Dallas County, TX between August 2021 and February 2022. All participants were referred from women's health clinics at the county's safety-net hospital system to an on-site drop-off childcare center by hospital staff who identified lack of childcare as a barrier to health care access. Participants were the primary caregiver for at least one child ≤age 13. Interviews were conducted in English or Spanish. We analyzed data using thematic content analysis.

**Results:**

We interviewed 22 participants (mean age 34); participants were adult women, had on average 3 children, and primarily identified as Hispanic or African American. Three interrelated themes emerged: disruptions in access, competing priorities, and exacerbated psychological distress.

**Conclusions:**

Findings demonstrate how low-income women with young children in a safety-net health system struggle to address their own health needs amid childcare and other household demands. Our study advances our understanding of childcare as a social domain of health, a necessary step to inform how we build structural support systems and drive policy interventions.

## Introduction

Childcare is an often-overlooked social domain of health. The American Pediatric Association Task Force on Child Poverty recognizes childcare as a basic social need,^1^ yet low-income families in the United States often lack reliable and safe childcare options.^2^ Furthermore, access to childcare remains one of the least studied socioeconomic factors influencing caregiver health upstream of the clinical encounter.^3–5^

Studies have shown that lack of childcare contributes to missed health care appointments for adult women.^6,7^ In a nationally representative study of adult women (*n*=2751), lack of childcare was the third most common logistical barrier (14%) to accessing health care services for themselves, behind inability to find time to go to a health care provider (24%) and inability to take time off work (23%).^6^ Like many social and structural barriers to health care, lack of childcare disproportionately affects women in low-income households in the United States,^2,6,7^ a population that experiences numerous other health disparities.^8^ Regarding the postpartum visit specifically, an appointment that is well known to be underutilized in the United States,^9^ qualitative research has shown that while most low-income women acknowledge the importance of it, many describe logistical barriers to attendance, including lack of childcare for their other children.^7^ While prior research has therefore linked lack of childcare to missed adult health care appointments in the United States, especially for low-income, underserved women, studies have stopped short of identifying additional health impacts.

Understanding the intersection between childcare and health is of increasing importance in the context of the COVID-19 pandemic given the pandemic's widespread impacts on physical health, mental health, and disruptions in childcare. In a study in the United Kingdom of 21 young parents' experiences, parents' mental health was substantially impacted by the pandemic due to worries about contracting COVID-19, social isolation, lack of support during perinatal appointments, and disruptions in perinatal care.^10^ In the United States, the pandemic increased material hardships for families with young children due to difficulty paying for basic needs, which correlated with rising parental emotional distress.^11,12^ Women with children have been especially affected because they often put caring for other family members ahead of their own mental health needs.^13^

Although much has been reported regarding women's worsening mental health during the pandemic,^10,13,14^ research is lacking regarding how women have dealt with their own health issues and coexisting childcare needs in the context of the COVID-19 pandemic. Our objective in this study was to understand in women's own words their experiences in coping with both childcare and health needs during the COVID-19 pandemic. We used qualitative methods to explore in detail how lack of childcare impacts the health of the caregiver.

## Methods

### Setting and participants

In Dallas County, TX, women's health providers at a safety-net health system, which provides care to low-income and uninsured residents of Dallas County, recognized that a substantial proportion of its patients seeking outpatient care reported needing childcare assistance even before the COVID-19 pandemic.^15^ In acknowledgment of this need, the health system partnered with a local community-based organization (CBO) to provide no-cost drop-off childcare services during health care appointments at an on-site childcare center that opened in November 2020.^16^ Participants were eligible for this study if they received care at the health system, identified themselves as a primary caregiver for at least one child ≤13 years, were English- or Spanish-speaking, and were referred to the on-site childcare center from the health system's women's health clinics because childcare needs were identified as a barrier to attending medical appointments.

### Recruitment

We identified 53 individuals eligible for the study. We sent a text message from the childcare center's business cell phone to all potential participants describing our study and providing the opportunity to opt out of a recruitment call. Participants designated in the electronic health record as Spanish speaking received the text message in Spanish. After several weeks, investigators began to call potential participants who had not opted out (*n*=52) to describe our study and assess eligibility and interest in participation. Of the 52 individuals called, 30 were able to be reached. One individual declined participation and 29 individuals indicated interest in participation. Of these 29 individuals, 7 were not able to be reached again for an interview. Between July 2021 and January 2022, 22 individuals consented to be interviewed. Participants provided verbal informed consent and received a $20 gift card as compensation for their participation in the study in accordance with the study protocol approved by UT Southwestern Medical Center's Institutional Review Board (STU-2021-0309).

### Data collection

We developed a semistructured interview guide informed by a literature review and study aims. We iteratively adapted the guide throughout the data collection process. Questions initially focused on the intersection of childcare and participant experiences with the health care system. Based on initial participant interviews and through discussions with the research team, we broadened questions to also explore the intersection of childcare, daily life, and participants' health overall. Interviews were conducted in participants' preferred language through telephone to reduce potential barriers due to transportation and internet access (K. Qualia, pers. comm., July 2021).

Investigators met regularly during data collection to review and discuss findings; recruitment of participants continued until repetition in findings (i.e., thematic saturation) was noted. Interviews were conducted between August 2021 and February 2022 and lasted an average of 25 min (range 12–34 min). We audiorecorded all interviews. English interviews were transcribed verbatim using NVivo transcription software,^17^ then edited for accuracy by the research team.

Spanish transcripts were transcribed verbatim using NVivo transcription software,^17^ edited for accuracy by the research team, and then translated into English by the health system's Language Services Department.

### Data analysis

S.J. kept detailed field notes throughout the research process, which were shared regularly during meetings with the research team and with partners at the childcare center. To allow for data immersion before coding,^18,19^ S.J. and C.S. individually reviewed the transcripts. Then, we conducted thematic content analysis using a combined deductive and inductive approach.^18^ Specifically, using a deductively driven codebook corresponding to the interview guide, team members met regularly to discuss, jointly code and refine codes and codebook definitions for the first 30% of transcripts. We also included an “emergent” code to categorize emerging concepts during the coding process that were not anticipated but represented important findings.

Once we finalized the codebook, two team members (S.J. and C.S.) each independently coded all remaining transcripts using NVivo.^17^ We decided upon final codes in meetings to resolve coding discrepancies. Once all transcripts were coded, we synthesized findings from each theme. Findings and representative quotes were interpreted with the entire research team and partners at the childcare center.

## Results

All 22 participants were adult women (mean age 34 years). Most participants identified as Hispanic or Latina (*n*=11) or Black or African American (*n*=9) and had an average of three children (Table 1).

**Table 1.** Participant Sociodemographic Characteristics, *n*=22

	***N*** (%)
Age (years), mean (range)	34 (24–49)
Race and ethnicity	
Black or African American	9 (41%)
Hispanic or Latina	11 (50%)
Non-Hispanic White	2 (9%)
Preferred language	
English	17 (77%)
Spanish	5 (23%)
No. of children, mean (range)	3 (1–5)

Several interrelated themes emerged from our analysis of participants' experiences of addressing health and childcare needs during the COVID-19 pandemic: disruptions in access, competing priorities, and exacerbated psychological distress.

### Disruptions in access

Many participants faced increased difficulty making in-person health care appointments for themselves during the pandemic.

*With the…changes of how many people that can come into the doctors' appointments…it's longer wait times, it's harder to get in. If you need a physical appointment, you literally sometimes have to go on up to the hospital, to the E.R., which raises your [COVID-19] exposure risk so much more. But you can't wait a week, two weeks, two months, two years to get into an appointment for a physical ailment. (Participant 16, 12/14/2021)*

Additionally, several participants lost health insurance during the pandemic because of job losses, which led to disruptions in health care.

*As soon as he [my husband] got the job, he got laid off because of the COVID situation. And then it just became a whole thing and we ended up missing out on enrolling [in health insurance]. (Participant 15, 11/18/2021)*

Most participants, however, noted that their major barrier to accessing health care during the pandemic was the increased difficulty in arranging childcare for their own medical appointments. Participants typically relied on relatives, friends, or neighbors to watch their children while they attended their medical appointments. Alternatively, participants would at times bring their children to their appointments with them or, for those who had school-aged children, arrange appointments during school hours. However, during the pandemic, participants noted more limited options for arranging childcare for medical appointments when visitor restriction policies were in place and their children were enrolled in remote learning at home.

*I really had no one [to watch my son]… like [when] my family had COVID and so he [my son] couldn't go over there [to stay with my family]. And then my friends, they couldn't watch him, they had to work. So there were different times when it was just very inconvenient for everybody. And so I just had to bring him with me and have him wait in the waiting area…or have him wait in the car. Or they was times I had to contemplate, I just have to miss the appointment or I have to reschedule or something or just be late and figure out everything… What do you want me to do? I'm a single parent. I have nowhere for him to go. (Participant 11, 10/26/2021)*

*My children were at home [during remote learning]…My daughter who is 9…I would have to bring her with me [to appointments]. And a lot of times I would get to the clinic and I would be turned away because I have her with me…Which became so straining, and it did delay a lot of things… I just kept getting turned away in every direction. (Participant 20, 1/11/2022)*

### Competing priorities

For many participants, family and household responsibilities were inextricably linked with their own health. Participants described managing these responsibilities as competing priorities, often with minimal support.

*I don't have anyone to rely on. Like I say, my mom is just now starting to help out. And my oldest is 14 years old, so I try to do it the best way I can possibly. I'm actually a year behind on a Pap smear…It's kind of hard because you try to put your health first, because in order for you to live a long and healthy life and be able to do for your kids, you have to worry about your health…But in my case, it's not possible right now with everything that's going on from transitioning to the domestic violence shelter to finally getting the kids in school and then working, it's just hectic. (Participant 19, 12/17/2021)*

*I had times where I would have a [menstrual] cycle and it would last months….I was bleeding heavily, where I was continuously messing my clothes. So being somewhere with your kids and trying to figure out how to take care of yourself and still give them what they need became hard. When I had to go home [after surgery] with the incision, I couldn't just pause not feeding them or pause not cleaning up. So I had to do those things still while taking care of my own self…There was times where I were in pain but I couldn't treat my pain because I had to see my kids that day, because the pills would make me sleepy and I couldn't take them because I needed to be up. (Participant 20, 1/11/2022)*

Even among those who had a partner, most participants described how they were still largely responsible for daily household and childcare tasks. This made it difficult to address their own health needs, especially when dealing with an acute health issue or a new health diagnosis.

*When I was diagnosed with diabetes and [the doctor] told me to do exercises, I wanted to go walking every day for at least 30 to 45 minutes. It's hard to balance work and then the kids and the cooking and cleaning and plus take a 30 minute walk every day. You don't have enough time. I wish I had like multiple persons, where part of me will clean and the other part of me will go take my walk… And it's still an issue now. But I mean, you just try to do the best that you can. I mean, I have their dad, but he's always at work…and the kids always come to me more than they do with him. I'm their go-to person. So, I mean, he's great, but he really doesn't help. (Participant 17, 12/16/2021)*

*We [mothers] do what we can and we try to function as best we can because you still have to take care of your kids, you still have to feed your kids, you still have to change diapers. And a lot of times you just do it while not feeling well.… If you cut back on certain things that aren't 100 percent necessary, you might not sweep the floor or fold laundry right away. But there's just some things you just can't get away from…Moms don't get sick days. (Participant 15, 11/18/2021)*

### Exacerbated psychological distress

The continuous need to tend to childcare while trying to address their own health needs was overwhelming and frustrating for many participants.

It's very frustrating and overwhelming as far as my own health because it's like, what's the point? I can't get to the appointment, or I can't reschedule in time or I can't get it arranged for when I need it because I can't get childcare or what have you. So it's just like very frustrating. It's like, f*** it. (Participant 16, 12/14/2021)

For some participants, managing childcare and their own health needs in the context of cumulative family and household stressors led to states of emotional distress.

*I mean, your kids still gotta eat and you still gotta take care of yourself. So sometimes that do become challenging, and I'm not gonna lie, it pushed me into a depression. And it made my anxiety, really, really become a new level, and it was really hard some days because you didn't know what to do and how to take care for yourself and care for them. And you just kinda become overwhelmed and go into overload. (Participant 20, 1/11/2022)*

*It was the worst pregnancy I ever had…just because of my diabetes…I had to check [my blood sugar level] like four or five times a day, staying constant [with] what I'm eating…My diabetes made it hard for me to enjoy my pregnancy…I already feel like it's a lot having two kids, dealing with school, dealing with trying to find a job, dealing with COVID and dealing with this, dealing with that. And then now I have to inject myself [with insulin] how many times a day, check what…I'm eating, stuff like that, it just made it a lot worse. (Participant 21, 1/20/2022)*

## Discussion

This qualitative study uniquely documents the health and childcare experiences of adult women with young children who received care at a safety-net hospital system in the United States during the COVID-19 pandemic. Findings indicate that lack of childcare options exacerbated disruptions in access to health care for participants. Additionally, childcare and traditional roles of motherhood were inextricably linked with participants' health, in that their health was both driving and impacted by caregiving and household needs, creating competing priorities that were difficult to meet. Finally, the interplay of childrearing and household demands with participants' health led to cumulative stressors and exacerbated psychological distress.

For participants in our study, access to health care was disrupted during the pandemic not only due to factors such as disruptions in health insurance and perceived longer waiting times, but overwhelmingly due to difficulty securing childcare for their own health appointments. This is important because disruptions in access to health care could potentially result in poorer health outcomes due to delayed or deferred care.^20^ Women in our study often relied on informal arrangements for childcare during medical appointments, which were not always dependable resources. This finding is consistent with prior studies that note the disproportionate use of informal arrangements for childcare among low-income families.^2^

For participants in our study during the pandemic, visitor restriction policies and remote learning exacerbated the limited childcare options that participants faced when arranging health care appointments. If their informal childcare arrangements fell through and they could no longer bring their children with them to their appointments, participants were forced to make difficult choices, such as canceling or missing their appointment or leaving their child unsupervised while attending their appointment.

This led to a sense of frustration and, at times, feeling that it was pointless to try to reschedule their own medical appointments, given that the same type of unreliable support would be required on subsequent occasions.

Childcare and health comprised competing priorities for participants, perhaps especially given that all participants were women with young children and were the primary caregivers in their households. Most participants noted they were primarily responsible for both routine household and childcare duties regardless of whether they worked or had a domestic partner. This finding highlights the disproportionate caregiving burden for women in the United States; despite the diversity of family compositions in modern U.S. society, traditional gender roles and expectations remain largely intact for women, especially for single mothers and heterosexual couples.^15,21–23^ The field would benefit from additional studies on how lack of childcare impacts health for women in queer households and households in which the partner is the primary childcare provider.

In our study, women described having minimal structural support to help address their many competing priorities, resulting in a struggle to balance their daily responsibilities while attempting to address their own health issues. Our findings demonstrate the need for structural support systems that address family and social context, especially for low-income women with young children, to assist patients in optimally managing their health.^15,24^ The health system-CBO collaboration to subsidize an on-site childcare center to which our participants were referred provides one example of a social care intervention that provides this additional support in the context of the health system.

Our finding that psychological distress was exacerbated among women struggling to balance childcare and household demands during the COVID-19 pandemic adds to the pandemic-era literature, which found that parents faced high levels of psychological distress and exhaustion in part due to prolonged social isolation and increased care demands.^15,25^ Our study further adds to these findings by highlighting how managing one's own physical health amid such stressors exacerbates emotional distress for mothers. Although participants in our study practiced coping skills such as time management or reprioritizing tasks, they still found it difficult to optimally manage their own health needs and felt overwhelmed. This is significant because it suggests that existing resources and strategies may be insufficient, and worse, unfairly place the onus on individuals to deal with cumulative stressors.^11,25–28^

Our findings again emphasize the need for additional structural support systems to reduce the burden on low-income women with young children. This includes not only providing early identification and referrals for mental health services but also advocating for expanded childcare assistance policies, such as childcare subsidies and paid family leave, at the state and federal levels.^15,24^

By underscoring the need for health systems to acknowledge childcare as a barrier to health for parents and caregivers, this study has important implications, particularly for women with young children in safety-net health systems. At minimum, clinics serving lower-income populations should ensure that logistical barriers to attending appointments are assessed, including the need and ability to arrange dependent care.^29^ Such a needs assessment by the health system in this study identified the lack of childcare as a major reason for missed and delayed appointments and led to the development of its partnership with a local CBO to provide no-cost on-site childcare during medical appointments. The health system's next steps will be to evaluate health care-related outcomes from this novel service, including whether the intervention has led to a reduction in missed or delayed appointments.

Limitations of our study include the small sample size and the use of convenience sampling, which may have introduced sampling bias such that participants may have been more likely to be included in our study based on unmeasured characteristics. Given that all women in our study served as the primary caregiver for their children, it is unclear how our findings on the intersection of childcare and health might apply to women who are not in primary caregiver roles. Strengths of this exploratory study include the generation of rich, descriptive data and the focus on a vulnerable population subject to health disparities.

## Conclusion

Women with young children who sought care at a U.S. safety-net health system during the COVID-19 pandemic experienced cumulative stressors, often with minimal support, making it difficult to optimally address their own health needs. This study adds to our understanding of how low-income women with young children may struggle to address their own health given competing priorities, including childcare and household demands. Follow-up studies could focus on exploring the interplay of family demands and medical and mental health issues for the birthing parent specifically in the postpartum period.

Our findings underscore the relevance and interconnectedness of family and social context to a patient's health and add to our understanding of childcare as a social domain of health for the caregiver. Understanding the health challenges and disparities associated with parents lacking childcare assistance is important to inform structural support systems and drive policy interventions. Future research should focus on quantifying the impact of childcare needs on health care outcomes (e.g., missed appointments and time to treatment) for parents and caregivers and evaluating interventions that seek to address these issues.

## Data Availability

All data generated and analyzed during this study are available from the corresponding author on reasonable request.

## Abbreviation Used

CBO: community-based organization
